# Effective Control of Molds Using a Combination of Nanoparticles

**DOI:** 10.1371/journal.pone.0169940

**Published:** 2017-01-25

**Authors:** Ariana Auyeung, Miguel Ángel Casillas-Santana, Gabriel Alejandro Martínez-Castañón, Yael N. Slavin, Wayne Zhao, Jason Asnis, Urs O. Häfeli, Horacio Bach

**Affiliations:** 1 Department of Medicine, Division of Infectious Diseases, University of British Columbia, Vancouver, BC, Canada; 2 York House School, Vancouver, BC, Canada; 3 Facultad de Estomatología, UASLP, San Luis Potosí, SLP, Mexico; 4 Faculty of Pharmaceutical Sciences, University of British Columbia, Vancouver, BC, Canada; Institute of Materials Science, GERMANY

## Abstract

Molds are filamentous fungi able to grow on a variety of surfaces, including constructed surfaces, food, rotten organic matter, and humid places. Mold growth is characterized by having an unpleasant odor in enclosed or non-ventilated places and a non-aesthetic appearance. They represent a health concern because of their ability to produce and release mycotoxins, compounds that are toxic to animals and humans. The aim of this study was to evaluate commercial nanoparticles (NPs) that can be used as an additive in coatings and paints to effectively control the growth of harmful molds. Four different NPs were screened for their antifungal activities against the mycotoxin producing mold strains *Aspergillus flavus* and *A*. *fumigatus*. The minimal inhibitory concentrations of the NPs were determined in broth media, whereas an agar diffusion test was used to assess the antimold activity on acrylic- and water-based paints. The cytotoxic activity and the inflammatory response of the NPs were also evaluated using the established human derived macrophage cell line THP-1. Results showed that a combination of mix metallic- and ZnO-NPs (50:10 μg/mL) effectively inhibited the fungal growth when exposed to fluorescent light. Neither cytotoxic effect nor inflammatory responses were recorded, suggesting that this combination can be safely used in humid or non-ventilated environments without any health concerns.

## Introduction

Molds are a large and taxonomically diverse group of filamentous fungi. They are important microorganisms with biotechnological benefits, such as the production of antibiotics, commercial enzymes, food, and beverages. They also perform important ecological functions by biodegrading organic matter. Molds are able to grow on a variety of surfaces, including buildings (indoors and outdoors), food, rotten organic matter, and in any humid place. Their growth is characterized by having an unpleasant odor and a non-aesthetic appearance [1]. Additionally, they have a negative public health concern as specific strains release toxins and spores to the environment generating allergy sensitivities and other diseases such as cancer [1]. *Aspergillus flavus* and *A*. *fumigatus* are two ubiquitous molds that produce mycotoxins, which are compounds toxic to animals and humans. *A*. *flavus* produces aflatoxin B1 and B2, aflatrem, and sterigmatocystin, whereas *A*. *fumigatus* produces fumagillin and gliotoxin [2]. These mycotoxins cause serious illnesses such as hepatotoxicity and cancer [3]. In the case of aflatoxin B1, the mechanism of toxicity is based on the alkylation of DNA through epoxy bonds [4], which causes cancer-inducing mutations [5]. *A*. *fumigatus’* toxins cause respiratory issues in immunosuppressed patients. Moreover, the growth of molds is prevalent in hospital environments, causing complications in Intensive Care Units post-surgery. Economical losses include the presence of *A*. *flavus* and *A*. *fumigatus* in storage rooms, which present ideal conditions for the fungal spores’ growth, resulting in significant food spoilage [6].

Molds are able to grow on painted walls because they can obtain nutrients by metabolizing compounds used in the fabrication of coatings and paints, such as plasticizers, which are added to these products to increase their fluidity. For example, it has been reported that *Aureobasidium pullulans* colonizes surfaces by metabolizing plasticizers present in paints [7].

To restrict mold proliferation, different antifungal compounds are added by manufacturing companies to coating and paints. For instance, organochlorines and quaternary ammonium salts are used to avoid microorganism growth, but they represent a health concern because of their toxicities.

Nanoparticles (NPs) have shown potent antimicrobial activities and have been extensively studied as an alternative to antibiotic agents [8,9]. NPs are highly reactive as a result of a high surface area-to-mass ratio and have been successfully used because of their optical, electrical, and chemical properties, which differ from their normal attributes at the macro scale. In addition, NP research is also focused on environmental care and improvement of human health [10].

Common elements used in the fabrication of NPs with antimicrobial activities include Ag and Au. Studies of the antifungal activities of these NPs are limited. However, NPs made from Ag, Cu, Ti in the form of TiO_2_, and ZnO have been shown to possess antifungal activities [8,11–14].

The purpose of this study was to evaluate the antimold activity of different commercial NPs, which can be used as an additive in coating formulations or paints to effectively control mold proliferation. We used two strains of *Aspergillus* as a fungal model and measured the antimold activity of different NPs. Since NPs may also be considered a health concern, we also assessed their potential cytotoxic and inflammatory effects using a human macrophage model.

## Materials and Methods

### NP source and characterization

The following commercial NPs were used in this study: metallic-NP (SolarCoat^®^, GreenWalls Bioengineering, Hong Kong), Ag- and Au-NPs (NN Labs, AR, USA), and ZnO-NPs (Meliorum Technologies, NY, USA).

NPs were characterized by scanning electron microscopy (FEI-Helios Nanolab 600, OR, USA) operated at 5.00 kV. The zeta potential and size distribution of aqueous suspensions were measured by dynamic light scattering (DLS) using a Malvern Zetasizer DTS 3000HS (Malvern, UK). To rule out a potential interference of the unknown ingredients of the commercial NP solutions, both metallic- and ZnO-NPs were washed by ultracentrifugation (40,000 rpm, 1h) using Sabouraud dextrose broth (SAB, BD, NJ, USA). Scanning electron microscopy (SEM) (JEOL 6510, Tokyo, Japan) was used for the elemental analysis and operated at an accelerating voltage of 15 kV, whereas transmission electron microscopy (TEM) was performed in a JEOL JEM-1230 (Tokyo, Japan) using an accelerating voltage of 100 kV.

### Fungal strains and culturing conditions

*A*. *flavus* (NRRL 3518) and *A*. *fumigatus* (ATCC 1022, VA, USA), both aflatoxin producers, were cultured in SAB at 30°C. The same medium supplemented with 1.5% agar was used for stock purposes at 4°C (solidified SAB). The growth of molds started from spores, which were obtained by gently rubbing the surface of sporulated cultures after growing the strains on solidified SAB for 1 week at the same temperature indicated above. Spores were stored at -20°C after the addition of 50% glycerol.

### Minimum inhibitory concentration (MIC) assay

The goal of this assay was to determine the lowest concentration of NPs, which inhibit the growth of the fungi. This experiment was performed in a 96-well microtiter plate and each well contained 5 μL of spore suspension (adjusted to 1x10^5^ spores/well), 20 μL of NP suspension, and 75 μL of SAB to total 100 μL/well. Untreated spores and amphotericin B (5 μg/mL, Sigma-Aldrich, MO, USA) were used as negative and positive controls, respectively. Plates were sealed with parafilm and incubated at room temperature for 72 h or until growth of the untreated spores (negative control) was observed. Plates were placed either in a dark place or continuously illuminated by a fluorescent light using a negatoscope (GE, IL, USA).

### Bauer-Kirby disk diffusion assay

This experiment was conducted to show the antifungal activities of the NPs when paints were supplemented with individual or combined NPs that showed antimold activity. Indoor acrylic-based (03651 Kitchen and Bath) and outdoor water-based (02203 Covercoat Flat) paints (Cloverdale Paint, Surrey, BC, Canada) were used to assess the NP antimold activity.

Spores were uniformly spread onto solidified SAB plates with inoculation loops and the plates were then left to dry for 10 min in a biological containment level 2. Then, autoclaved-sterilized filter disks (6 mm diameter) were coated with either water-based (white) or acrylic-based (purple) commercial paints, and NPs were deposited and mixed onto these disks. Plates were placed in the dark or under fluorescent light on a negatoscope for 2 weeks and the zones of inhibition were measured. Amphotericin B was used as a positive control.

### Cytotoxicity analysis

The human monocyte THP-1 cell line (ATCC TIB-202) was used to evaluate the toxicity levels of metallic- and ZnO-NPs as well as the combination of both NPs. THP-1 cells were grown as monocytes in RPMI medium supplemented with heat deactivated 10% fetal calf serum (Hyclone, GE, IL, USA), and a mixture of penicillin (100 μg/mL) and streptomycin (100 U/mL) (StemCell, Vancouver, BC, Canada). THP-1 cells were incubated at 37°C in a humid atmosphere of 5% CO_2_. Cells (1x10^4^ cells/well) were activated the night before of the experiment by the addition of 40 ng/mL phorbol myristate acetate (PMA, Sigma). The next day, the medium was aspirated and replaced with fresh media without antibiotics and exposed to the NPs for 24 h using the same incubation temperature as stated above. Thereafter, 3-(4,5-dimethylthiazol-2-yl)-5-(3-carboxymethoxyphenyl)-2-(4-sulfophenyl)-2H-tetrazolium (MTS, Promega, WI, USA) was added to the wells and the optical density of the plates was read at 450 nm using a plate reader (Epoch, Tecan, Männedorf, Switzerland). Staurosporine (1 μg/mL, Sigma) and untreated cells were used as positive and negative controls, respectively. The cytotoxic activities were performed without exposure to the light to mimic the environment once the NPs are penetrating into the body.

### Immunological response

Activated THP-1 cells were also used to determine the immunological response when these cells were incubated with the NPs for 24 h. Supernatants were collected and used to measure the levels of the pro-inflammatory cytokines interleukin 6 (IL-6) and tumor necrosis factor α (TNF-α) using commercial kits (BD). Lipopolysaccharide from *E*. *coli* (1 μg/mL, Sigma) and untreated cells were used as positive and negative controls, respectively.

### Statistical analysis

Experiments were performed in triplicate and the significance of the results was analyzed by a *t*-test analysis. P<0.05 was considered as significant.

## Results

### NP characterization

A preliminary test was performed to screen the antifungal activity of NPs against the two strains of *Aspergillus* used in this study (see below). Results showed that only metallic- and ZnO-NPs showed a potent antifungal activity and these NPs were used during the rest of this study. These NPs were characterized by zeta potential, size distribution, and the dispersity index (Table 1).

**Table 1:** Characterization of the NPs.

Nanoparticle	Zeta potential (mV)	Size (nm)	Dispersity index
**Metallic**	**-13.9 ± 4.84**	**7**	**0.487**
**ZnO**	**-17.4 ± 3.52**	**477**	**0.413**

**Fig 1:**
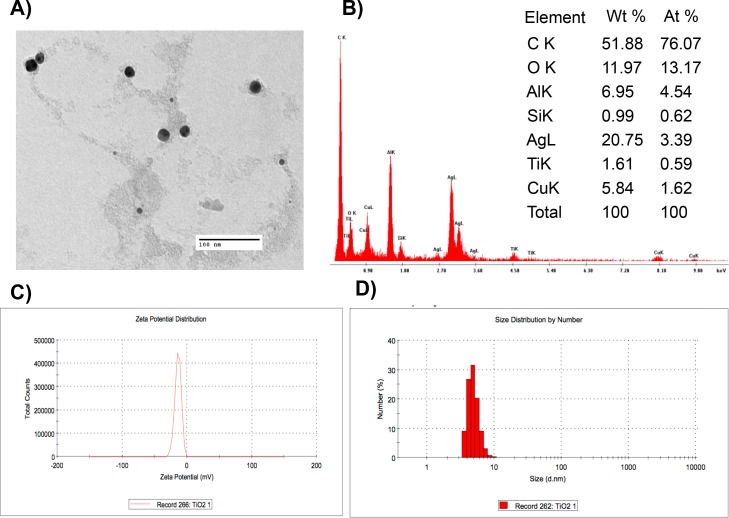
Characterization of metallic-NPs. Metallic-NPs were characterized using (A) TEM, (B) element analysis by SEM, (C) zeta potential, and (D) size distribution.

**Fig 2:**
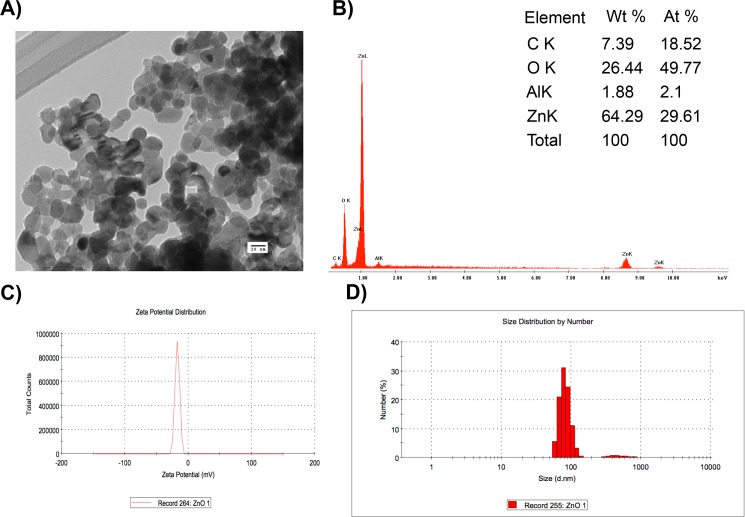
Characterization of ZnO-NPs using (A) TEM, (B) element analysis by SEM, (C) zeta potential, and (D) size distribution.

Electron microscope images showed that the distribution size of metallic-NPs was homogeneous, whereas a heterogeneous distribution was observed in ZnO-NPs (Figs 1 and 2). Elemental analysis using SEM confirmed the heterogeneity of metallic-NPs (Fig 1B), whereas the presence of ZnO was confirmed in the ZnO-NPs (Fig 2B) as shown in the table included in both figures.

### MIC measurement

Individual and combined NPs were tested against both strains of *Aspergillus*. Most of the individual NPs tested showed a MIC of 100 μg/mL except for Ag-NPs that showed a MIC of 10 μg/mL, but only against *A*. *flavus*. Au-NPs were inactive against both fungal strains (Table 2).

**Table 2:** Antifungal activity of NPs against *A*. *flavus* and *A*. *fumigatus* expressed as MICs (μg/mL).

Strain	Nanoparticle					
	Ag	Au	Metallic	ZnO	Metallic:Ag	Metallic:ZnO
***A*. *flavus***	R	R	100	20	R	50:10
***A*. *fumigatus***	10	R	100	20	R	50:10

R, resistant

Based on these preliminary results, only metallic- and ZnO-NPs were selected for combination testing. Surprisingly, an enhanced effect was observed at a combination of metallic:ZnO-NPs (50:10 μg/mL), but was absent when metallic:Ag-NPs (50:5 μg/mL) was tested. This enhancement was manifested by a reduction of 50% of the MICs of both NPs (Table 2). Additionally, plates stored in the dark showed no antifungal activity (data not shown).

### Antifungal activity of paint embedded with NPs

**Fig 3:**
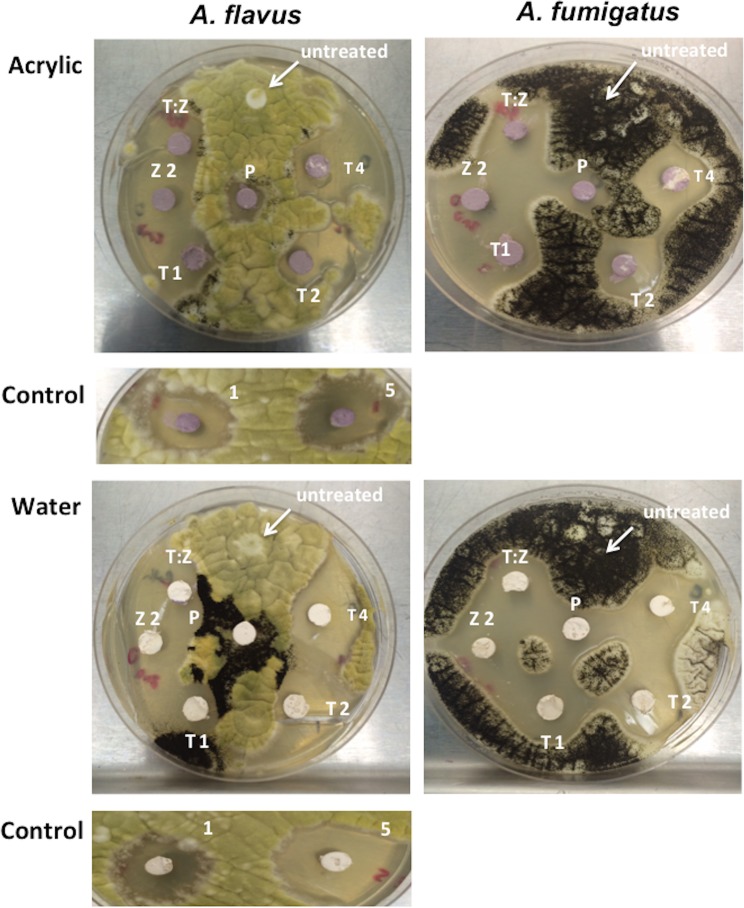
Growth inhibition of ZnO- and metallic-NPs against *Aspergillus flavus* and *A*. *fumigatus*. Concentrations of metallic-NPs are T1 = 100 μg/mL, T2 = 200 μg/mL, and T4 = 400 μg/mL. Z2 is 20 μg/mL of ZnO-NPs. T:Z represents the ratio of metallic:ZnO-NPs (50:10 μg/mL). P = untreated paint, Control = amphotericin at 1 μg/mL and 5 μg/mL.

**Fig 4:**
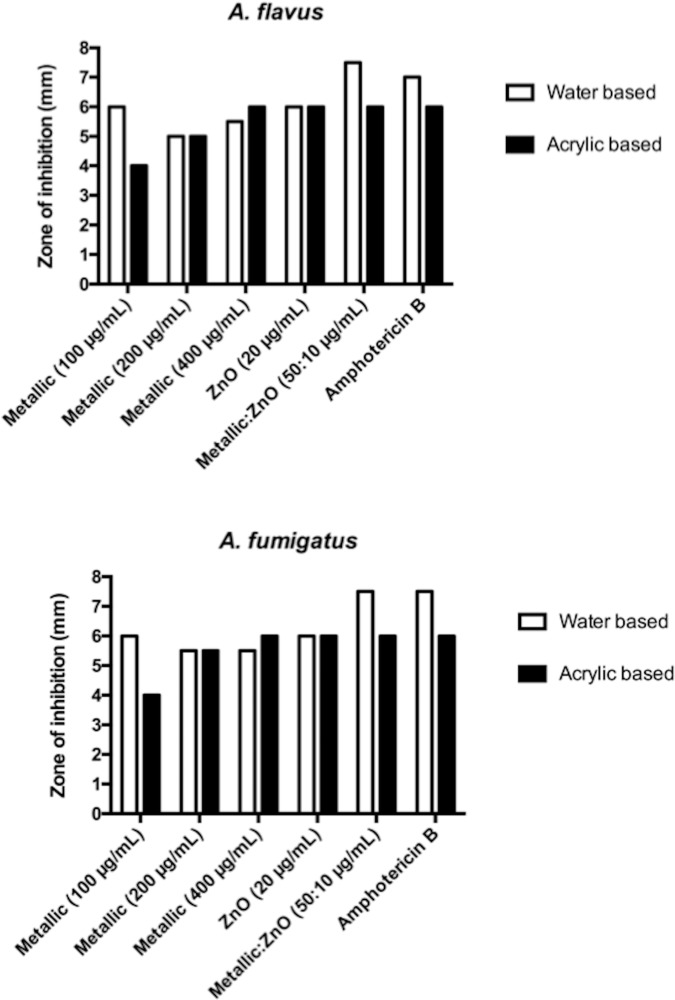
Measurement of the zone of inhibition (in mm) of ZnO- and metallic-NPs against *Aspergillus flavus* and *A*. *fumigatus*.

The antifungal activity was tested in paints by mixing the NPs with water- or acrylic-based paints. The zone of inhibition was evaluated after 2 weeks. As in the case of the MIC assay, only the plates exposed to the fluorescent light showed antimold activity. Measurements of these zones of inhibition clearly showed antifungal activity (Figs 3 and 4) of the NPs, whereas a combination of metallic- and ZnO-NPs showed the highest activity similar to amphotericin used as a positive control.

### Cytotoxic and inflammatory activities

**Fig 5:**
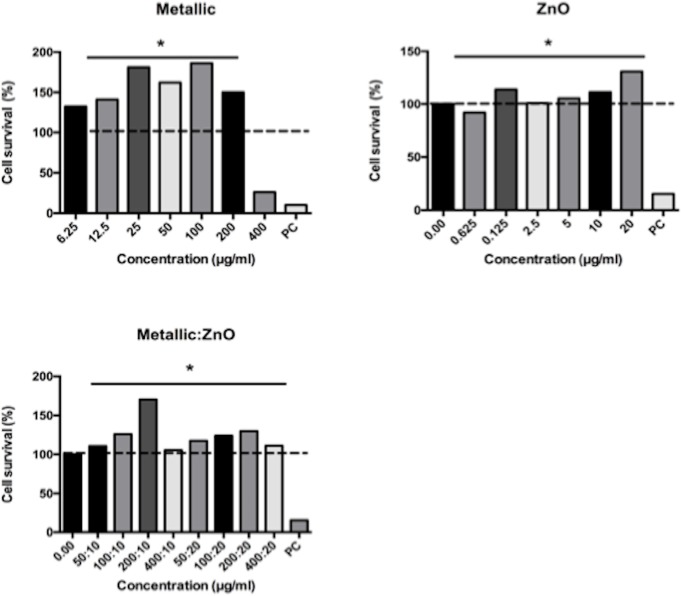
Cytotoxic activity of ZnO- and metallic-NPs exposed to human macrophages. The cell survival (%) was normalized to the untreated cell values. PC = positive control. The dashed line represents 100% cell survival. The significance was compared to the positive control.

Metallic-NPs were toxic at a concentration of 400 μg/mL. However, ZnO- and the combination of metallic:ZnO-NPs showed no toxicity even at the high concentrations (Fig 5).

In addition, an inflammatory response was not significant because of undetectable levels of the cytokines in the supernatant of the culture (data not shown).

## Discussion

In this study we tested the antifungal activity of NPs against two mold strains of *Aspergillus*. We chose to test commercial NPs because their availability in industrial amounts. Thus, commercial available NPs, including Ag-, Au-, metallic- and ZnO-NPs were chosen for a preliminary antifungal activity screening. After the screening, we decided to continue our study with metallic- and ZnO-NPs, which showed a potent activity against both strains.

The characterization of the NPs showed that metallic-NPs had a homogeneous size, whereas a broader size distribution was observed in ZnO-NPs (Figs 1 and 2). Although the values measured for the zeta potential indicate a tendency for the NP to agglomerate, this concern is not expected to be an issues because a homogeneous dispersion will be produced as a result of the addition of surfactants to paints and coatings during their fabrication [15]. These surfactants are necessary to emulsify the binder and to disperse the pigment, providing stability to the paints. Common surfactants used in the industry include anionic and non-ionic surfactants, such as sodium dodecyl sulfate and alkyl phenol ethoxylates with concentrations up to 6% by weight [16].

Results of the antifungal activity showed that both metallic- and ZnO-NPs killed both fungal strains at MICs of 100 μg/mL, indicating that these NPs effectively control the proliferation of molds. Interestingly, this antifungal activity was obtained only under fluorescent light, but not when the plates were incubated in the dark, suggesting that the light activates these NPs. The metallic-NPs used in this study contained a mixture of elements, including Ti and O_2_. Although we are unable to determine the presence of TiO_2_ in the NPs, it has been shown that TiO_2_ generates highly toxic free radicals when illuminated with ultraviolet light [17]. This process is based on the movement of an increased number of electrons from the valence band (energy filled bands) to the conduction band (higher energy of unfilled bands) forming more electron-hole pairs upon excitation with UV. These electrons can react with O_2_, H_2_O, or CO_2_ surrounding the fungi in a continuous photocatalytic process, generating toxic reactive oxygen species (ROS), such as superoxide and hydroxyl radicals. Other contributors to the generation of ROS are Ag and Cu also present in the metallic-NPs. The radicals generated by these processes can induce detrimental life threatening effects to any cell by damaging protein, lipids, and DNA [17,18].

In the case of Ag, a potent antifungal activity (IC_80_ = 1–4 μg/mL) was measured when 3 nm Ag-NPs were exposed to *Trichophyton mentagrophytes*, a pathogenic filamentous fungus [19]. Other filamentous fungi susceptible to Ag-NPs were clinical isolates of *Aspergillus*, *Alternaria*, and *Fusarium* isolated from fungal keratitis. In this study, MIC_90_ values of approximately 1 μg/mL were measured [20].

Similar photocatalytic processes and the generation of ROS were also observed with ZnO-NPs [21,22]. Surprisingly, in our study, the antifungal activity of a combination of both metallic- and ZnO-NPs at lower concentrations (50:10 μg/mL) was similar to the activity of each individual NP in higher concentrations (100 μg/mL). This significant enhancement in the killing activity of this combination can be explained by an additive effect of ROS, such as hydroxyl radicals and singlet oxygen, generated by both NPs upon illumination [23,24]. In this regard, it has been reported that concentrations of 443 μM and 277 μM were measured when TiO_2_ and ZnO-NPs were illuminated, respectively [25]. In another study, a similar enhancement of the photocatalytic activity of a mixture of TiO_2_ and ZnO nanofibers were obtained, which was superior than the individual nanocomposites [26]. Authors concluded that this enhancement can be the result of a high efficiency in the separation of electron–hole pairs generated by the light and based on the interaction among the nanocomposites [26]. Taking together, the antifungal activity of the NPs tested in this study is a direct function of their photocatalytic activity, as the NPs cannot diffuse from the paint towards the solid agar.

The antifungal activity of ZnO-NPs measured in our study was superior to other studies that have reported in a variety of fungal strains. For example, a decrease of colony growth ranging between 60–90% was recorded when *Botrytis cinerea* and *Penicillium expansum* were exposed to ZnO-NP concentrations of 6 mmol/L [11], whereas activities >100 μg/mL were measured against the mold strains *A*. *niger* and *Rhizopus stolonifer* when exposed to the same type of NPs [27]. Other studies reported either the use of higher concentrations (>1.25 mg/mL) of Zn-NPs to control the growth of *A*. *niger* [28] or weak activities such as 11.5% and 5.3% growth inhibition registered against *Alternaria alternata* and *Chaetomium expansum*, respectively [29]. Similar weak antifungal activities (<75% growth inhibition) with ZnO-NPS were reported against the phytopathogenic strains *Pythium debarynum* and *Sclerotium rolfsii* [30].

Other studies reported a very potent activity of Ag-NPs against yeast, which are unicellular fungi belonging to a different taxonomy group and different morphologies. In this regard, MICs in a range of 0.052–0.84 μg/ml were reported against *Candida albicans*, *C*. *parapsilosis*, and *C*. *tropicalis*, whereas MICs up to 5 μg/mL were reported against *Sacharomyces cerevisiae* and *Trichosporon beigelii* [31,32].

Studies reporting the use of NPs in coating or paints are very scarce. However, the antifungal activity of TiO_2_- and ZnO-NPs individually incorporated to different paints was assessed against the molds *A*. *niger* and *P*. *chrysogenum*. Results of this study reported that only the ZnO-NPs at a pigment volume concentration of 5% limited the growth of the molds on surfaces [33]. Results of this study cannot be compared to our results because the different dimensions used to express the results.

To determine whether the NPs used in our study are toxic, we measured their cytotoxicity upon exposure to THP-1 cells, an established model of human-derived macrophages. Exposure of individual or combined NPs showed no cytotoxic effects at concentrations up to 400 μg/mL except for metallic-NP, which was toxic at this high concentration. Similar results were obtained when ZnO-NPs were exposed to normal peripheral blood mononuclear cells [34]. However, cytotoxic effects were measured when ZnO-NPs at concentrations as low as 10 μg/mL and 14–20 μg/mL were exposed to bronquial epithelial cells and HepG2 cells, respectively [35,36]. The analysis of these results suggests that the toxicity of NPs depends directly on several physical characteristics such as size, shape, charge of the NPs and on the phagocytic and ROS production capabilities (activation of cellular stress) [37,38]. In the case of TiO_2_-NPs, it has been reported that these NPs were not toxic to HeLa cells at the maximal concentration of 120 μg/mL tested [39], whereas 70–80% and ~80% cell survival were registered when rat kidney cells and mouse fibroblasts were exposed to 100 μg/mL and 600 μg/mL of TiO_2_-NPs, respectively [40,41]. However, discrepancies with these results were found when human amnion epithelial cells showed cytotoxicity when exposed to TiO_2_-NPs at 10 μg/mL [42].

The ZnO-NPs were not able to elicit an immune response in our study because no release of significant amounts of the pro-inflammatory cytokines IL-6 and TNF-α were measured, suggesting that exposure to the studied NPs will not induce an inflammatory response. Similar negative results were reported for the same cytokines we reported when C3A hepatocytes were treated with ZnO-NPs [43]. Discrepancies with our results were reported when aortic endothelial cells were exposed to ZnO-NPs at concentration >10 μg/mL and murine macrophages (RAW cells) at concentrations of 0.3 μg/mL [44,45]. The differences in the capability of mounting an inflammatory response can be attributed, as mentioned earlier, to the internalization capacity of the NPs, which depends on the physical characteristics as mentioned earlier. In our study, a heterogeneous size population of 477 nm of ZnO-NPs was measured, which may difficult the internalization of the NPs into the mammalian cells, and the inflammatory response is not activated properly.

In the case of TiO_2_-NPs also discrepancies in the reported studies can be found. For example, an increase in the inflammasome activity was measured when murine dendritic cells were exposed to these NPs [46], whereas no cytokine changes were observed when a range of different TiO_2_-NPs (7–94 nm) were exposed to C3A hepatocytes [47].

## Conclusions

We tested commercial metallic- and ZnO-NPs to control the growth of molds. Both NPs showed antifungal activity under continuous illumination with fluorescent light. An enhancement in the combined NPs was observed when halves of the MIC concentrations were combined. The addition of such NPs to paints or coating will reduce the mold proliferation with a consequent drop in the release of mycotoxins and allergens to the environment. Then, paint or coating formulations containing metallic- and ZnO- NPs will limit the growth of molds on surfaces, specifically in humid and non-ventilated environments with very few potential health concerns such as cytotoxicity and immunological responses. We also conclude that a release of NPs from the formulation is not expected because of the presence of binder and surfactant in the paint formulation, which will keep the NPs on the surfaces.
